# Muscle–Tumor Crosstalk in Exercise Oncology: Exercise-Induced Myokines and Cancer Cachexia

**DOI:** 10.3390/cancers18142343

**Published:** 2026-07-20

**Authors:** Amirhossein Ahmadi Hekmatikar, Francesco Bettariga, Álvaro López Llorente, Diego Fernández-Lázaro, Katsuhiko Suzuki, D. Maryama Awang Daud

**Affiliations:** 1 Department of Physical Education and Sport Sciences, Faculty of Humanities, Tarbiat Modares University, Tehran 10600, Iran; 2 School of Human Performance, Rehabilitation and Population Health, INSIGHT Research Institute, University of Technology Sydney, Sydney, NSW 2021, Australia; francesco.bettariga@uts.edu.au; 3 Exercise Medicine Research Institute, Edith Cowan University, Joondalup, WA 6027, Australia; 4 Department of Sports Medicine, Burgos Burpellet BH UCI Pro Team, 09007 Burgos, Spain; 5 Neurobiology Research Group, Faculty of Medicine, University of Valladolid, 47005 Valladolid, Spain; diego.fernandez.lazaro@uva.es; 6 Area of Histology, Faculty of Health Sciences, University of Valladolid, Campus at Soria, 42004 Soria, Spain; 7 Consolidated Research Group ENSADE (Envejecimiento, Neurociencia, Salud y Desarrollo), León Biosanitary Research Institute (IBIOLEÓN), 24071 León, Spain; 8 Faculty of Sport Sciences, Waseda University, Tokorozawa 359-1192, Japan; katsu.suzu@waseda.jp; 9 Health Through Exercise and Active Living (HEAL) Research Unit, Faculty of Medicine and Health Sciences, Universiti Malaysia Sabah, Kota Kinabalu 88400, Sabah, Malaysia; 10 Department of Biomedical Sciences, Faculty of Medicine and Health Sciences, Universiti Malaysia Sabah, Kota Kinabalu 88450, Sabah, Malaysia

**Keywords:** solid tumors, cancer, exercise, myokines, exerkines

## Abstract

Exercise-induced myokines, such as IL-6, irisin, and myostatin, play crucial roles in mediating systemic adaptations and modulating tumor progression. This narrative review synthesizes evidence on how regular moderate-intensity exercise affects the myokine network within the tumor microenvironment (TME) of solid tumors. Results from the 86 included studies indicate that structured training (aerobic: 45–65% VO_2_ max; resistance: 45–70% 1RM) enhances the expression of beneficial myokines that support immune surveillance, reduce inflammation, and improve muscle–tumor cross-talk. IL-6 demonstrates a dual modulatory effect on inflammation and immunity; irisin contributes to tumor apoptosis, while myostatin downregulation supports muscle integrity and metabolic balance. Evidence suggests that exercise-induced alterations in these factors may influence tumor angiogenesis and immune infiltration, offering potential therapeutic implications. Overall, tailored exercise regimens represent a promising non-pharmacological strategy to optimize myokine signaling in cancer management. Further human studies are needed to confirm these mechanistic effects and define optimal exercise protocols. While existing data suggest that structured exercise may beneficially modulate myokine profiles and thereby influence tumor biology, these conclusions are based on limited qualitative evidence rather than quantitative synthesis. Further well-controlled clinical studies are required to confirm causality and define optimal exercise prescriptions for oncology settings.

## Simple Summary

Exercise is increasingly recognized as a powerful adjunct in cancer care, not only by improving fitness and reducing fatigue, but also by reshaping the biological dialog between skeletal muscle and tumors. In this structured narrative review, we synthesize current evidence on exercise-induced myokines and their roles in tumor biology and cancer cachexia. We focus on key mediators such as myostatin, follistatin, irisin, interleukin-6, fibroblast growth factor 21, and myonectin, highlighting how their effects depend on exercise type, intensity, timing, and the tumor microenvironment. Importantly, these signals can have dual and context-dependent actions: they may suppress inflammation, support immune surveillance, and preserve muscle mass, but under certain conditions they may also promote tumor growth, angiogenesis, or immune evasion. Overall, the evidence suggests that exercise can beneficially modulate muscle–tumor crosstalk, yet the biological effects of specific myokines remain complex and cancer-type dependent. A deeper mechanistic understanding is needed to translate these insights into personalized exercise strategies for patients with cancer and cachexia.

## 1. Introduction

In the face of alarming reports about the rising global prevalence of cancer, it is disheartening to see that this disease has continued to increase and has now become the leading cause of death [1]. If current trends persist, all types of cancer are projected to double in incidence by 2070 compared to 2020 [1]. Despite the advancements in cancer screening and detection methods, there are still numerous individuals being diagnosed with this devastating illness [2]. Solid tumors (STs), including breast, colorectal, lung, and pancreatic cancers, are characterized by complex biology and substantial therapeutic resistance, which pose major challenges for effective treatment [3,4]. STs are characterized by their intricate nature, setting them apart from other forms of cancer. These tumors exhibit resistance to drug treatments, aggressive behavior, high intelligence, and angiogenic properties. Moreover, the inflammatory physiology of STs can induce detrimental alterations in surrounding tissues, such as muscle, resulting in cachexia [5,6]. Cancer-related muscle wasting (cachexia) is tightly linked to dysregulated myokine signaling, and growing evidence suggests that exercise-induced production/release of myokines may counteract tumor-driven catabolic pathways [7,8]. Despite the existence of treatment options such as surgery, targeted drug delivery, chemotherapy, and immunotherapy, all of which carry intricate and high financial, psychosocial, and physical burdens, there is no definitive strategy or approach for combating ST [9,10].

Exerkines encompass a diverse array of signaling molecules released during acute and/or regular exercise, exerting their effects through endocrine, paracrine, and/or autocrine pathways [11,12]. These exerkines originate from various sources, including skeletal muscle (myokines), the heart (cardiokines), liver (hepatokines), white adipose tissue (adipokines), brown adipose tissue (batokines), and neurons (neurokines) [11,12]. Among exerkines, myokines have garnered the most attention in oncology research due to their critical roles in immune regulation, metabolic homeostasis, and inter-organ communication [11,12,13]. However, to accurately interpret their roles, a clear conceptual distinction must be made between myokines and classic cytokines. While classic cytokines are primarily synthesized by immune cells in response to inflammation or pathology, myokines are defined as peptides specifically expressed and released by contracting skeletal muscle fibers in response to mechanical and metabolic stress. A prime example of this distinction is interleukin-6 (IL-6); while immune-derived IL-6 acts as a chronic, pro-inflammatory cytokine that promotes tumor progression, muscle wasting, and cachexia, exercise-induced muscle-derived IL-6 functions transiently as a metabolic myokine, exerting systemic anti-inflammatory effects independent of classic inflammatory pathways [11,12,13]. Beyond IL-6, other muscle-derived factors play pivotal roles. Irisin has been implicated in the browning of white adipose tissue, a process associated with the induction of apoptosis in certain tumor cells [14]. Similarly, myostatin—known as a negative regulator of muscle growth—has been shown to decrease with regular exercise, potentially mitigating cancer-related muscle wasting [11,12,13]. Also, emerging evidence suggests that myokines modulate tumor biology through pathways such as JAK/STAT (IL-6), AMPK-PGC1α (irisin), and PI3K/Akt (myostatin), although the exact downstream effects in different tumor types remain under investigation [11,12,13]. However, the exact mechanisms of how exercise and tumors interact still require further investigation, as reported by these researchers. This hypothesis raises an important question: Can the lactate released through exercise impact tumor growth [14]?

Despite its rich history rooted largely in fitness, exercise has often taken a back seat in the field of disease treatment and is seen as a secondary strategy [15]. However, understanding the fundamental and impactful mechanisms of exercise physiology in relation to diseases can greatly contribute to the development of more practical strategies, even though it remains a challenging task that researchers are actively exploring [12]. While the clinical benefits of exercise during cancer are still being studied, initial findings suggest its potential in reducing disease severity, mitigating the side effects of chemotherapy, and managing negative physiological changes [16,17]. However, the profound effects of exercise on the tumor environment are not yet fully understood. In line with the positive effects of exercise on the tumor mechanism, studies reported that exercise can lead to a decrease in tumor size by reducing inflammation (increasing anti-inflammatory cytokines) [18,19]. In fact, in one study, researchers [11] highlighted the crucial role of exercise-induced exerkines in modulating antitumor immunity, improving tumor-related metabolic regulation, and attenuating pathways involved in cancer-associated muscle wasting. However, multiple studies have indicated that myokines, which are proteins secreted by muscles, are the primary exerkines influenced by unrestricted exercise [20,21]. Additionally, in cancers affecting skeletal muscle or involving muscle infiltration, the muscle tissue is often among the first to exhibit pathological changes related to altered myokine signaling [22,23,24]. The positive effects encompass enhancements in muscle strength and improvements in immune system functionality, particularly at low to moderate intensities. Conversely, high-intensity exerkines may lead to a decline in immune system function. Nonetheless, the existing knowledge concerning the relationship between exerkines and cancer lacks specificity. Hence, the objective of this study is to ascertain the current understanding of exerkines and their impact on cancer tumors.

Cancer-related muscle wasting (cachexia) represents one of the most clinically significant consequences of solid tumors and is tightly linked to dysregulated myokine signaling. Tumor-driven inflammation and metabolic stress alter key myokines such as IL-6, myostatin, FGF-21, irisin, and myonectin, leading to accelerated proteolysis and impaired muscle regeneration. Because exercise is a potent regulator of these molecules, understanding how myokines mediate muscle–tumor crosstalk is essential for clarifying both the beneficial and potentially harmful effects of exercise in cancer settings. Therefore, muscle wasting constitutes a central biological pathway in this review and forms a critical framework for interpreting the impact of exercise-induced production/release of myokines on tumor physiology.

Despite notable progress in uncovering the clinical benefits of exercise in oncology, current evidence lacks the necessary mechanistic depth to guide translational application. Previous reviews have primarily addressed general associations between exercise and cancer outcomes, without fully delineating how distinct exercise-induced myokines—such as IL-6, irisin, and the less-studied myonectin—orchestrate signaling within the tumor microenvironment (TME) [25]. Furthermore, critical molecular cascades, including JAK/STAT, AMPK–PGC1α, and PI3K/Akt, have not been systematically integrated into a cohesive framework that links exercise dose and modality to tumor physiology. To bridge these gaps, this review provides a molecularly grounded synthesis that moves beyond descriptive associations. Unlike previous works that focus on isolated pathways or primarily beneficial outcomes, we offer a more balanced translational perspective that considers context-dependent and potentially adverse signaling. Moreover, we position myokine signaling within the broader exercise–TME axis, while critically addressing the limited standardization of exercise prescription as a major barrier to clinical translation. By uniting physiological context, mechanistic insight, and a rigorous critique of methodological gaps, this review advances the field toward a more precise framework for exercise oncology.

## 2. Materials and Methods

### 2.1. Search Strategy

This study is a structured narrative review, conducted between April and July 2025, whose objective was to evaluate the impact between exercise, myokines, and the tumor microenvironment. The literature search was conducted using the electronic databases Medline (PubMed), Sci-ELO, and Cochrane Library Plus. The search strategy included terms related to exercise, cancer, exerkines and its outcomes, as well as a combination of these terms using the MeSH index: Myokines, Cardiokines, Hepatokines, Adipokines, Batokines, Neurokines, Solid Tumors, Cancer, Exercise, Myokines, Exerkines. Myostatin, Follistatin, Irisin, Interleukin-6 and Myonectin were linked using the Boolean operators “AND” and “OR.”

Additional records were identified through a snowball search by screening the reference lists of studies eligible for full-text review. Titles and abstracts were reviewed independently by two reviewers (A.A.H. and D.F.-L.) to identify duplicates and assess eligibility.

### 2.2. Inclusion and Exclusion Criteria

Study quality was considered descriptively during the data-extraction stage. Although this review did not employ a formal risk-of-bias scoring system—given its structured narrative design—each included article was evaluated for methodological clarity, peer-review status, and completeness of reporting (sample characteristics, exercise intensity, and outcome measures). Studies presenting major design limitations or insufficient reporting were discussed cautiously and were not used to make definitive quantitative conclusions. Variability among study findings, particularly the divergent responses of some myokines to similar exercise modalities, likely reflects differences in study quality, participant characteristics, exercise protocols, and methodological approaches rather than true biological contradictions. The following inclusion criteria were applied to the selected articles: (1) full-text access; (2) review articles, clinical trials, observational studies, experimental studies, clinical experience reports, or case reports; (3) identification of the relationship between exercise, exerkines, and the tumor microenvironment; (4) articles published since the databases’ inception, with no date restrictions; (5) languages were limited to English, German, French, Italian, Spanish, and Portuguese.

Studies were excluded if they did not meet one or more of the predefined inclusion criteria, were not directly related to the interaction between exercise-induced exerkines and the tumor microenvironment, lacked sufficient methodological information, were not available in full text, or corresponded to publication types outside the predefined eligibility criteria of this review (e.g., editorials, conference abstracts, or opinion papers).

In this review, “acute exercise” refers to a single, isolated bout of exercise, whereas “chronic exercise” refers to repeated exercise sessions over several weeks or months that induce physiological training adaptations.

In total, 603 unique records were identified through database and reference searches. After duplicate removal and screening of titles and abstracts, 126 full-text articles were assessed for eligibility, of which 86 met all inclusion criteria and were ultimately incorporated into the review.

The literature selection process followed a sequential strategy including database searching, duplicate removal, title and abstract screening, and full-text eligibility assessment. Although this study was designed as a structured narrative review rather than a systematic review, these procedures were implemented to maximize transparency, reproducibility, and methodological rigor during study selection.

In this review, heterogeneous study designs were included because the aim of this structured narrative review was to integrate evidence across both mechanistic and clinical domains. Given the multidimensional nature of exercise-induced myokine responses and their interactions with the tumor microenvironment, inclusion of both preclinical and human studies was considered essential to provide a comprehensive overview of the current state of knowledge.

## 3. Results

### 3.1. The Relationship Between Myokines and Tumors

To enhance clarity, Section 3 was reorganized to integrate both aspects of the analysis—exercise–myokine interactions and myokine–tumor relationships—within unified subsections for each major myokine. This approach allows the reader to follow how specific exercise stimuli modulate individual myokines and, in turn, how these myokines influence tumor growth, angiogenesis, and immune responses. Consequently, Section 3.1, Section 3.2, Section 3.3, Section 3.4, Section 3.5, Section 3.6, Section 3.7, Section 3.8, Section 3.9 and Section 3.10 are now organized by myokine type rather than by exercise or tumor separately, providing a more coherent mechanistic narrative.

### 3.2. Mechanistic Interplay Between Myokines and Cancer Biology

Exercise induces dynamic changes in the skeletal muscle secretome, resulting in marked alterations in the magnitude, pattern, and physiological relevance of myokine release compared with resting conditions. While basal secretion contributes to metabolic homeostasis, muscle contraction during exercise triggers rapid and often several-fold increases in specific myokines, including IL-6, irisin, and myostatin. More than 600 myokines have been identified through secretome analyses of human myocyte cultures [26]. However, only a subset has been functionally characterized, and several have been consistently identified as contraction-induced molecules, highlighting the central role of skeletal muscle activity in regulating systemic signaling [26,27]. This distinction between resting and exercise-induced myokine profiles provides the mechanistic framework for understanding their interactions with the tumor microenvironment discussed in the following sections.

### 3.3. Tumor Growth and Angiogenesis

In the field of clinical research, an interesting connection between myokines and the complex world of the tumor microenvironment has been revealed. The findings have illuminated the profound impact myokines can have on the physiological state of tumors [24,28]. It is widely recognized that the relationship between myokines and cancerous tumors is reciprocal, with muscles being among the organs profoundly influenced during cancer development and tumor formation [28,29,30]. Consequently, it becomes increasingly apparent that the tumor environment can induce behavioral alterations in myokines, while myokines, in turn, can elicit physiological changes within the tumor environment [28,29,31,32,33,34,35,36,37,38]. This tumor-derived influence triggers adaptive changes in myokine behavior; however, the direction and magnitude of these changes depend strongly on whether the stimulus involves acute or regular exercise. Acute exercise typically induces transient elevations in myokines such as IL-6, irisin, and FGF-21, whereas long-term training is more often associated with reductions in myostatin and IL-6 and a stabilization or moderate increase in follistatin. In contrast, tumor-driven conditions tend to upregulate myostatin, downregulate follistatin, increase IL-6, irisin, and FGF-21, and may suppress myonectin levels [32,33,34,35,36,37,38]. These behavioral shifts, in turn, trigger inflammation, impaired immune system function, and muscle atrophy [29,31].

The influence of myokines on tumor angiogenesis is complex. IL-6, which is elevated both in tumors and during exercise, plays a dual role. It can support tumor angiogenesis in some contexts, yet when induced through controlled, moderate-intensity exercise, IL-6 has also been shown in preclinical studies to contribute to vascular normalization and improved immune infiltration [39]. This duality underscores the importance of contextual interpretation, particularly regarding acute vs. chronic exercise. Transient spikes in IL-6 following high-intensity exercise may differ from adaptive IL-6 signaling resulting from sustained training. It is important to emphasize that the effects of individual myokines are highly context-dependent—varying not only according to tumor type but also to the modality, intensity, and duration of exercise. For example, IL-6 may exhibit anti-tumoral properties in breast and lung cancer where it enhances immune infiltration and apoptosis, yet it can promote pro-tumoral inflammation in colorectal or liver malignancies under chronic stress conditions. Similarly, myostatin and irisin demonstrate opposite trends depending on whether the exercise is acute or chronic. To capture these complexities, the following subsections are organized by myokine type, each providing an integrated discussion of its bidirectional behavior in different tumor contexts and its responsiveness to various exercise regimens.

### 3.4. Myostatin

Myostatin, one of the transforming growth factor-beta (TGF-β) family, assumes a crucial role in regulating skeletal muscle mass by negatively influencing its growth [40]. Among its primary functions is its ability to induce behavioral changes in muscle hypertrophy. When myostatin levels are low, muscle growth ensues, whereas high expression of myostatin leads to muscle atrophy, a phenomenon commonly observed in cancer-induced cachexia [11,41]. Cancerous tumors are well-known for their aggressive nature [42,43], often resulting in muscle atrophy accompanied by behavioral alterations in myostatin, transforming individuals into a lean state that impedes their ability to engage in exercise [42,43,44]. This process typically begins with sarcopenia—an early loss of muscle mass and function—and may advance to cancer cachexia, a systemic condition characterized not only by severe muscle wasting but also by multi-organ metabolic disruption, inflammation, and a markedly elevated risk of morbidity and mortality [44]. Considering that cancerous tumors induce the overexpression of myostatin by signaling to the muscles, this can be seen as an initial step in the behavioral changes in myokines in alignment with the tumor’s physiology [45]. Furthermore, experimental animal studies have shown that overexpression of myostatin promotes the migration of tumor-associated macrophages into the tumor microenvironment, thereby facilitating cancer progression [46]. Additionally, recent findings indicate that Mstn (myostatin) can support the proliferation of tumor cells while simultaneously contributing to muscle atrophy in adjacent tissues, indicating a dual detrimental role of myostatin within the tumor microenvironment [47]. In addition, it has recently been found that myostatin is expressed in various tumor tissues and human cancer cells, and the tumor environment uses the produced myostatin to its advantage for the growth and atrophy of surrounding tumor tissues [47].

### 3.5. Follistatin

Follistatin and myostatin perform opposing functions. While myostatin inhibits muscle growth, follistatin emerges as a myokine that counteracts the effects of myostatin [48,49]. Imbued with autocrine origins, follistatin is a glycoprotein predominantly secreted by muscle tissue. Amplifying its expression fosters muscle growth, while dampening it triggers muscle atrophy [48,49]. Astonishingly, follistatin also appears to wield considerable influence over tumor physiology [50]. Janik et al. (2019) showed follistatin’s ability to induce angiogenesis in the vicinity of tumor tissue [50]. The underlying mechanism for this angiogenic effect and subsequent tumor growth is associated with a novel signaling pathway. Follistatin, as a natural antagonist of activin A, impedes the interaction between activin A and its receptors, namely activin type I and II, leading to the internalization and degradation of the activin complex via endocytosis and lysosomal pathways [51,52]. Adding to the intrigue, multiple studies have unveiled the involvement of follistatin (FST) in tumorigenesis, metastasis, and angiogenesis of solid tumors. FST achieves this by engaging with activin and BMPs (bone morphogenetic proteins), thereby inducing pathophysiological effects [51,52]. Any intervention that modulates the behavior of these two myokines holds the potential to both benefit and harm the tumor simultaneously.

### 3.6. Irisin

Irisin is a key adipomyokine that coordinates multiple signaling pathways of metabolic processes. Generated from the proteolytic hydrolysis of fibronectin type III domain-containing protein 5 (FNDC5), irisin emerges as a newly discovered cytokine induced by exercise [53]. Present throughout the human body, irisin plays a multifaceted role in diverse physiological functions, including the browning of white adipose tissue, enhancement of insulin resistance, improvement of cognitive function, and regulation of bone metabolism [54]. However, recent research has revealed a strong and intimate association between irisin levels and tumors. Interestingly, investigations have found that irisin concentrations surge in all types of tumor tissues compared to their normal counterparts [55]. Furthermore, it has been established that irisin plays a pivotal role in the initiation, progression, and metastasis of diverse tumors, positioning it as a potential target for tumor diagnosis and treatment [55]. Interestingly, in the context of solid tumors, it has been observed that cancer patients exhibit significantly reduced serum levels of irisin compared to healthy individuals, suggesting a tumor suppressive role for this adipomyokine [55,56,57]. A specific study focusing on breast cancer patients revealed a substantial decrease in serum irisin levels among individuals with breast cancer. Experimental studies, predominantly conducted in preclinical models, have shown a close correlation between irisin levels and the incidence of breast cancer, positioning irisin as an independent predictor of this malignancy [56]. Evidence from a limited number of studies has suggested that higher circulating irisin levels may be associated with a substantially lower risk of breast cancer. One study reported that each unit increase in circulating irisin was associated with an approximately 90% reduction in breast cancer risk; however, this finding should be interpreted with caution until confirmed by additional clinical studies [55,56,57]. Nevertheless, despite the emerging evidence indicating the inhibitory effects of irisin on tumor proliferation and growth, conflicting findings have also been reported. Some studies have suggested that irisin levels increase in breast, thyroid, and lung cancer, thereby facilitating tumor growth and proliferation [58,59]. These divergent outcomes may stem from the diverse tissues responsible for irisin release and the varying quantities thereof, influenced by both local and systemic production [53,58,59].

### 3.7. Main Sources of IL-6 Beyond Skeletal Muscle

Interleukin 6 (IL-6) is a cytokine that plays a pivotal role in the intricate web of immunity and inflammation regulation. Its influence extends far and wide, exerting diverse effects on various cell types and tissues [60]. However, when it comes to cancer, IL-6 assumes a predominant role in fueling tumor growth. The dynamic interplay between IL-6, its receptor-activated JAKs, and the consequential induction/activation of STAT3 through tyrosine phosphorylation, followed by transcription of target genes, emerges as a critical factor in the process of carcinogenesis [61,62]. Experimental studies have shown that IL-6 can promote an inflammatory response within the tumor microenvironment while concurrently suppressing anti-tumor immune function [63]. Furthermore, IL-6 can promote metastasis in certain types of cancer by enhancing inflammatory pathways and tumor cell migration [64]. Consequently, the role of IL-6 in tumor biology is poised as a potent predictor of aggressive malignancy. Although skeletal muscle has long been recognized as the principal origin of circulating IL-6 during exercise, additional tissues also participate in IL-6 synthesis and release, highlighting its systemic and multifunctional nature. Adipose tissue serves as a major reservoir of IL-6 under sedentary or inflammatory conditions. Adipocytes and resident macrophages within adipose depots secrete IL-6 in response to metabolic stress [64]. Regular exercise downregulates this source by reducing adipose inflammation and restoring insulin sensitivity, thereby lowering baseline IL-6 concentrations. Endothelial cells and monocytes/macrophages release IL-6 when exposed to exercise-induced shear stress or catecholamine surges. This transient endothelial IL-6 response promotes angiogenesis and tissue repair and contributes to the acute spike observed immediately after exercise. Hepatic tissue acts both as a target and a secondary producer of IL-6. Muscle-derived IL-6 stimulates hepatic signaling through the JAK/STAT3-SOCS3 axis, enhancing acute-phase protein production (CRP, fibrinogen) [64]. Conversely, chronic training may suppress hepatic IL-6 transcription, reflecting long-term anti-inflammatory adaptation. Cardiac and neural tissues can express IL-6 under oxidative or high-load conditions [64]. In cardiomyocytes, IL-6 triggers PGC-1α-mediated mitochondrial remodeling, and in neurons, IL-6 influences hypothalamic regulation of energy balance and stress response. Altogether, these findings underline that exercise stimulates IL-6 release through coordinated activation of muscular, adipose, vascular, hepatic, and neural sources [64]. Therefore, IL-6 should be viewed not simply as a muscle-only myokine, but as a multi-organ exerkine integrating immune, metabolic, and regenerative communication across tissues [64].

### 3.8. Fibroblast Growth Factor 21

The fibroblast growth factor (FGF) family comprises 22 members, which can be divided into conventional FGFs and hormone-like FGFs [65]. FGFs play a vital role in various cellular processes, including cell growth, differentiation, angiogenesis, embryonic development, wound healing, and metabolic regulation [65]. Among these FGFs, fibroblast growth factor 21 (FGF-21) stands out as a multifunctional peptide hormone, known as a myokine, that participates in several endocrine functions, including the regulation of glucose transport and lipid metabolism [66]. While FGF-21 has demonstrated beneficial effects on health, studies have discovered its significant impact on tumor growth. Specifically, increased levels of FGF-21 in the bloodstream have been shown to promote tumor growth and enhance aggressiveness [66]. Interestingly, different studies have presented conflicting results, suggesting that FGF-21 may contribute to the growth and proliferation of cancerous tumors. Angiogenesis may play a role in this process [67,68,69,70]. Recent research has indicated that FGF-21 plays a role in invasion and metabolic disorders in thyroid cancer, with elevated levels of FGF-21 in thyroid cancer patients potentially indicating tumor progression [67]. On the other hand, a contradictory study suggests that preclinical studies have suggested that FGF-21 inhibits the growth of prostate cancer cells and induces apoptosis through autophagy [71]. Tumor cells are metabolically active and susceptible to nutrient deprivation, and FGF-21 may influence their behavior [71]. It appears that FGF-21 can suppress tumor cell proliferation, migration, and invasiveness while promoting apoptosis [71]. Moreover, overexpressing FGF-21 has been found to significantly inhibit the proliferation and migration of tumor cells induced by high glucose levels [71]. However, due to conflicting results, it is not possible to definitively determine the effectiveness of FGF-21 against tumors. Nevertheless, its suppressive effects seem to be more prominent.

### 3.9. Myonectin

Myonectin (CTRP15), a well-characterized protein, belongs to the C1q/TNF (CTRP) family [72]. While predominantly found in muscle, it is also present in circulation, particularly associated with nutritional metabolism [72]. Myonectin has garnered attention for its potential role in regulating metabolic processes and its association with various health conditions [72]. Research suggests that myonectin may contribute to glucose and lipid metabolism. It has been shown to enhance insulin sensitivity, aiding in maintaining optimal blood sugar levels [72]. Moreover, myonectin actively participates in the regulation of lipid metabolism, encompassing the breakdown and utilization of fatty acids. This multifaceted protein also exhibits promising anti-inflammatory properties [72]. It effectively inhibits the production of pro-inflammatory molecules while promoting the release of anti-inflammatory substances [72]. This anti-inflammatory effect could potentially safeguard against metabolic disorders and chronic inflammation-related diseases like obesity and cardiovascular disease [72]. As myonectin is secreted by skeletal muscle tissue, a myokine, it may indirectly influence tumor growth and progression through its impact on metabolism and inflammation [73]. Nevertheless, the precise effects of myonectin on tumors remain unclear and require further investigation (Table 1).

The findings summarized in Table 1 illustrate the complex and context-dependent roles of exercise-induced myokines in cancer biology. Rather than exerting uniformly beneficial or detrimental effects, individual myokines may influence tumor growth, angiogenesis, immune regulation, and cancer cachexia through distinct molecular pathways that vary according to the tumor type and biological context. The following sections discuss the current evidence regarding the interactions between exercise, myokine signaling, and tumor progression, highlighting both the therapeutic potential and the remaining translational challenges.

The importance of physical activity in maintaining a healthy lifestyle and preventing cancer has long been recognized [74,75]. A study by Park et al., 2023 has found that following exercise, blood tests, a significant portion of the exercise-responsive proteins detected in circulation were identified as myokines, indicating their central role in exercise-induced systemic signaling [76]. However, current studies now focus on implementing exercise protocols to manage cancer [77,78]. To develop effective training protocols for cancer patients, it is crucial to comprehend the fundamental physiological and tumoral mechanisms, as the situation differs with the presence of a tumor [30,79]. In this context, a study conducted by Chow et al. in 2022 [12], published in the journal *Nature Reviews Endocrinology*, elucidates the relationship between exercise, diseases, and exerkines. The researchers emphasized that our understanding of the physiological systems connected with disease is still limited, and exerkines can help researchers gain a better understanding of how exercise influences systemic inflammation, immune responses, and tumor progression [10]. Another study by Hekmatikar et al. in 2023 [14] demonstrated the profound impact of exerkines on cancer, with myokines appearing to play a prominent role in cancer cachexia. Exercise releases exerkines from muscle tissue, which modulate muscle atrophy and weight loss, contributing to disease reduction and control [11]. Therefore, considering the specific behavior of myokines during tumor formation, this section addresses the importance of one of the most critical exerkines, namely myokines, in exploring the complex field of exercise and cancer in the context of solid tumors. Furthermore, exercise may be a valuable intervention for the management of cancer cachexia, as it can help counteract muscle wasting by increasing protein synthesis, improving insulin sensitivity, and having anti-inflammatory effects. These effects provide increases in muscle mass, strength, and overall quality of life, among others [80].

### 3.10. Myostatin Response to Exercise

When it comes to investigating the acute myokine responses to resistance exercise, we found contradictory and limited findings. Despite this complexity, resistance training plays a significant role in changing myokine dynamics. Several studies have reached different conclusions regarding myostatin levels following high-intensity resistance training at 80–95% of one-repetition maximum (1RM). Some have reported unchanged levels [81,82], while others have observed a reduction [83,84,85,86]. MacKenzie et al. (2023) conducted a study indicating that a resistance training session at 30% of RM does not affect serum myostatin levels, whereas an 80% RM intensity can lead to a decrease in myostatin [84]. MacKenzie et al. (2013) found that acute resistance exercise reduces myostatin signaling by activating the TGFβ inhibitor Notch, resulting in a decrease in myostatin transcriptional activity [87]. Similarly, Gonzalo et al. (2013) demonstrated that resistance exercise consisting of three sets of 12 repetitions at 80–85% 1RM could lower myostatin [88]. Another study revealed that resistance training with an intensity of 70–80% of 1RM, involving 3–4 sets and 6 repetitions, can lead to a decrease in myostatin [89]. Additionally, acute resistance training comprising 4 sets of 10 repetitions at 90% 1RM was shown to impact myostatin expression [85]. Despite the predominance of high-intensity exercises in the examination of resistance exercises, a study indicated that low-intensity resistance exercises (60% of 1RM) with blood flow restriction can also effectively reduce myostatin [90]. Consistent with the reviewed studies, other investigations have shown that resistance training at intensities ranging from 80 to 90% 1RM, with 10–12 repetitions and 3–4 sets, can decrease myostatin [81,91]. The magnitude of myostatin reduction resulting from resistance training in the reviewed studies varied between 35% and 55% [81,83,84,85,86,87,88,89,90]. Conversely, McIntosh et al. (2023) reported contradictory results, stating that resistance training at 30% RM and 80% RM does not alter myostatin levels [82]. In relation to other forms of exercise, it appears that aerobic exercise at 70% of VO_2_ max in a single session can lead to a decrease in myostatin levels, which may persist for up to 4 h post-exercise [92]. Furthermore, supporting these findings, high-intensity interval training at 90% of HRmax was shown to immediately reduce myostatin levels, with the reduction lasting for 2 and 6 h post-exercise [93]. While the majority of studies indicate a decrease in myostatin, there are also studies reporting an increase. One study found that high-intensity intermittent exercise (Wingate test) can increase myostatin immediately after exercise, potentially attributed to decreased or absent IGF-1 secretion and increased IL-6 [94].

When it comes to the response of myokines to chronic effects, numerous studies have been conducted, most of which have reported a decrease in myostatin. A study by Bagheri et al. (2019) investigated the impact of 8 weeks of upper body, lower body, or combined resistance training on the ratio of follistatin to myostatin [94]. The researchers concluded that both upper body and lower body resistance training, with an intensity of 50–80% of 1RM, could lead to a decrease in myostatin. However, it is important to note that the volume of activated muscle mass plays a role in the changes observed in myostatin levels [94]. Another study found that individuals who engaged in resistance training three times a week for 12 weeks, at an intensity of 40–90% of 1RM, experienced a decrease in myostatin. This reduction was observed in women with breast cancer [95]. In a separate study by Hulmi et al., the effects of 21 weeks of resistance training on myostatin levels were investigated. The results indicated that 21 weeks of resistance training can lead to a significant decrease in myostatin. The intensity of resistance training in this study ranged from 40 to 85% of 1RM, with 3–5 sets and 12–20 repetitions [96]. Consistent with the studies reviewed in this section, numerous other studies have reported significant reductions in myostatin levels with resistance training. These studies typically involved an intensity between 50 and 85% or 90% of 1RM, 3–5 sets, and 10–15 repetitions, conducted over 8 to 16 weeks [94,95,97,98,99]. However, it is worth noting that the magnitude of the myostatin decrease varied between 50 and 70% in these studies [94,95,97,98,99]. It is important to consider that different studies may present varying opinions, but it appears that the activation of the mTOR and IGF-1 pathways can influence myostatin and contribute to its reduction [94,95,97,98,99].

Regarding other types of exercises, it seems that they also align with the reduction in myostatin observed in resistance exercises. For instance, one study found that aerobic exercise with an intensity of 40–55% of VO_2_ peak over 8 weeks can lower myostatin levels [100]. In a study by Konopka et al. (2018), aerobic exercise with an intensity of 50–65% of VO_2_ max, lasting 30–50 min for 12 weeks, was found to reduce myostatin [101]. Furthermore, another study demonstrated that continuous aerobic exercise at an intensity of 50–60% of reserve heart rate for 20–30 min during the first 4 weeks, gradually increasing to more than 60% of VO_2_ max and up to 80% of VO_2_ max for 50 min, can also result in myostatin reduction [102]. In relation to aerobic exercises, it has been discovered that engaging in these exercises at intensities ranging from a maximum of 50–65% of VO_2_ max or 40–55% of VO_2_ peak, with a duration of 30 min over a period of 6 to 12 weeks, can lead to a decrease in myostatin levels [76,100,101,102,103,104,105,106,107].

### 3.11. Behavior of Exercise-Released Myostatin with Tumors

Myostatin (which inhibits muscle growth) is implicated in oncologic cachexia by promoting muscle loss through inhibiting muscle hypertrophy and activating muscle degradation pathways. This process contributes to the muscle wasting observed in oncologic cachexia, making myostatin a potential therapeutic target to combat muscle wasting in cancer patients. Myostatin inhibitors are being investigated to counteract its effects and promote muscle growth in this setting [108]. Therefore, decreased myostatin is detrimental to tumor viability. In this way, a synergistic combination of strength training and aerobic activity has been shown to play an important role in reducing myostatin levels. Nevertheless, the resistance training component has a greater impact on these transformative changes. While immediate or acute responses to both resistance and aerobic exercises exhibit a decline in myostatin levels [81,82,83,84,85,86,87,88,89,90,93,94], the focus is primarily on the long-term adaptations observed in chronic responses [81,82,83,84,85,86,87,88,89,90,93,94,100,109,110]. Resistance training has the ability to reduce myostatin levels [100,109,110]. This significant drop is expected to impede the tumor’s access to myostatin, potentially reducing its influence on tumor growth.

### 3.12. Follistatin Response to Exercise

Just like myostatin, follistatin is another myokine that has captured the attention of researchers for its relevant molecular changes during exercise. While there is not an abundance of short-term research specifically focused on follistatin, we will utilize the available literature. Generally, studies employing resistance training as an intervention have indicated that a training protocol consisting of 70–90% of one’s maximum weight, 8–15 repetitions, and 3–4 sets can effectively increase follistatin levels [83,111,112,113,114]. Surprisingly, some of these studies reported a remarkable 25–43% surge in follistatin levels following short-term resistance training [83,111,112,113,114].

Regarding the aerobic exercise on follistatin research, Kon et al. in 2022 revealed that acute endurance exercise by running on a 150 to 300 m per minute for a duration of 35 min stimulates the production of follistatin [115]. Kon et al. in 2021 also demonstrated that a high-intensity SIT (Sprint Interval Training) cycling session, consisting of four 30 s maximal efforts with a 4 min recovery between sets, also triggers follistatin production [116]. Considering the constraints faced in previous studies Kon et al. in 2022 emphasized the importance of enquiring the reasons underlying the increase of follistatin during acute exercise responses [115]. It seems that an increase in growth hormone (GH) and/or insulin may play a pivotal role in elevating follistatin levels [117,118,119]. However, due to the scarcity of available studies, further research is necessary to arrive at more definitive conclusions regarding the long-term response to exercise.

To obtain more accurate insights into the changes in follistatin resulting from training, exploring the long-term responses is important. A study by Tolouei Azar et al. in 2019 revealed that individuals who engaged in eight different exercises, performing three sets of ten repetitions at 70% of their 1RM over an eight-week period, three times per week, showed significant changes in their follistatin levels [120]. Another study indicated that participating in resistance training for eight weeks, with intensities ranging from 60% to 95% of 1RM, employing 3 to 4 sets and completing 10 to 12 repetitions, led to an elevation in follistatin [121]. A study by Motahari Rad et al. in 2023 showed that engaging in resistance training with intensities ranging from 50% to 95% of 1RM, performing 10 to 15 repetitions across 3 to 4 sets over a twelve-week timeframe, results in an increase in follistatin [122]. Additionally, Arabzadeh et al. in 2020 highlighted that six weeks of resistance training, incorporating intensities between 60% and 85% of 1RM and 12 to 15 repetitions, contributes to an upsurge in myostatin [123]. Furthermore, multiple studies aligned with the aforementioned research have consistently demonstrated that resistance training with intensities ranging from 60% to 90% of 1RM, performing ten to fifteen repetitions, and encompassing a minimum of three sets and a maximum of five sets, effectively boost follistatin levels by approximately 30% to 60% [107,124,125,126,127,128].

In parallel with resistance training interventions, an extensive review of studies examining aerobic training unveiled that regular aerobic exercises lead to an elevation in follistatin [129,130,131,132]. It appears that engaging in aerobic activities with intensities ranging from 45% to 55% of VO_2_ max, lasting between 30 and 60 min, at least three times per week for a duration of 6 to 12 weeks, can contribute to a significant 12% to 25% increase in follistatin [129,130,131,132].

### 3.13. Behavior of Exercise-Released Follistatin with Tumors

Follistatin has the potential to promote angiogenesis, which can be both beneficial and detrimental in cancer patients [50]. Its proangiogenic effect is beneficial, as it can improve blood flow and accelerate healing and muscle recovery after injury by stimulating blood vessel growth [50].

However, in cancer, angiogenesis is crucial for tumor growth and metastasis. Tumors require blood flow to grow beyond a certain size and spread to other parts of the body. Therefore, follistatin’s ability to promote angiogenesis may contribute to tumor progression and potentially worsen the prognosis of cancer patients [133]. Follistatin levels are elevated in some cancers, and this elevation is associated with more aggressive tumors and worse prognoses. For example, in thymic epithelial tumors, elevated serum levels of follistatin were correlated with tumor stage and recurrence and associated with increased microvascular density, an indicator of angiogenesis [50]. Despite its potential negative impact on cancer, follistatin also has potential as a therapeutic target. Understanding how follistatin affects tumor angiogenesis could lead to new cancer treatment strategies, such as the development of therapies that inhibit follistatin’s proangiogenic effects or the use of follistatin-targeted therapies [50].

Both resistance and aerobic exercise, whether performed acutely (in a single session) or chronically (over a period), have been shown to increase follistatin levels in the body [134]. Increased follistatin due to exercise is thought to contribute to muscle hypertrophy (growth) and potentially other metabolic benefits, as follistatin may also influence glucose uptake and energy expenditure [134]. Resistance exercise tends to have a more pronounced effect on follistatin levels compared to aerobic exercise in an animal model [50] or in sedentary young women [134]. Thus, resistance training could produce significant increases in follistatin, while aerobic training may have a more moderate impact [83,107,111,112,113,114,115,117,118,119,120,121,124,125,126,127,128,129,130,131,132]. Consequently, considering the evidence that physical activity, particularly resistance training, can lead to heightened follistatin levels; it appears that this surge in follistatin may not be suitable for cancer patients with tumors.

### 3.14. Irisin Response to Exercise

Irisin, a recently discovered adipomyokine, has emerged as an exciting new myokine. A study by Raisi et al. in 2013 revealed that resistance training sessions with an intensity of 60–80% of one-repetition maximum (1RM), comprising 6–12 repetitions and 3–5 sets, lead to a notable increase in irisin levels [135]. Another study demonstrated the effectiveness of resistance training involving 70–95% 1RM, 6–8 repetitions, and 3 sets in boosting irisin production [136]. Similarly, Tsuchiya et al. in 2015 found that resistance training with 65% 1RM, 12 repetitions, and 3–4 sets can elicit an increase in irisin [137]. Several other studies have supported these findings, indicating that resistance exercises within the range of 65–90% 1RM, 8–12 repetitions, and 3–5 sets significantly contribute to the elevation of plasma irisin levels [138,139,140,141]. However, it is important to note that the magnitude of the irisin response to resistance training varies between 9% and 23%, depending on factors such as the specific muscles engaged and the intensity of the exercise [135,136,137,138,139,140,141,142].

Interestingly, a conflicting study reported that resistance training at 60% 1RM has no effect on irisin secretion [143]. The researchers suggested that this lack of change was due to the number and intensity of the training sessions [143]. On the other hand, an intervention study exploring aerobic exercise revealed that a cycling session lasting 50 min at 80% of VO_2_ max induce an increase in irisin levels, which can persist for up to 4 h’ post-exercise [144]. A meta-analysis further supported these findings, concluding that irisin concentration significantly rises immediately after acute exercise in adults [145]. Additionally, several studies have demonstrated the significant role of anaerobic exercises performed at intensities equal to or greater than 80% VO_2_ max or 80% of maximum HR [137,139,146,147,148] as well as aerobic exercises at 65% VO_2_ max, in promoting immediate irisin elevation [139]. The reported increases in irisin levels across these studies ranged from 26% to 35%, with the intensity of the training potentially contributing to the observed variations [137,139,146,147,148].

This section encompasses a comprehensive review of various studies examining the effect of resistance and aerobic training, without differentiating between the two. A study conducted by Kim et al. in 2016 investigated the influence of resistance and aerobic training on irisin levels [149]. The findings revealed that resistance training, comprising 8 weeks of 2–3 weekly sessions, with an intensity ranging from 65% to 80% of one-repetition maximum (1RM) and 10–12 repetitions, led to an increase in irisin. Additionally, aerobic exercise, involving 5 days per week of treadmill running and cycling for 60 min, at an intensity of 65–80% of individual maximum heart rate (HRmax), proved effective in elevating irisin levels [149]. However, the increase in irisin was more pronounced in the aerobic training group compared to the resistance training group. Another significant study by Shabani et al. in 2018 examined the effects of resistance training, aerobic training, and combined training on irisin [97]. The resistance training protocol, based on the recommendations of the American College of Sports Medicine (ACSM), entailed 3–4 sets, 8–12 repetitions, 30–60 s of rest between sets, 2–3 min of rest between exercises, and an intensity ranging from 60% to 75% of 1RM. Aerobic training, on the other hand, was performed at an intensity ranging from 60% to 75% of HRmax, while combined training involved a combination of resistance and aerobic exercises. The researchers reported that 4 weeks of resistance and aerobic exercise did not yield a significant increase in irisin, whereas combined exercises had a notable effect on irisin elevation [97]. Moreover, studies incorporating resistance training demonstrated an elevation in plasma irisin levels among subjects engaging in resistance training for 8 weeks, with an intensity of 55% of 1RM, 10–12 repetitions, and 3 weekly sessions [150]. These findings were supported by Zhao et al. in 2017, who found that 12 weeks of resistance training, performed at an intensity of 65–75% of 1RM, led to an increase in irisin levels [151]. Consistently, studies investigating resistance training interventions reported significant increases in irisin levels when utilizing intensities ranging from 55% to 85% of 1RM, repetitions ranging from 8 to 15, and sets ranging from 3 to 5, conducted over a span of 6–12 weeks [152,153,154,155]. The magnitude of the reported increase in irisin levels resulting from resistance training varied from 10% to 28% across these studies [151,152,153,154,155]. Furthermore, numerous studies explored the effects of aerobic and anaerobic exercises, all of which consistently reported heightened irisin levels. These investigations demonstrated that aerobic exercises performed at intensities ranging from 50% to 65% of VO_2_ max or 60–75% of HRmax, as well as anaerobic exercises conducted at intensities between 70% and 85% of VO_2_ max or 80–95% of HRmax, over a duration of 5–14 weeks, with exercise sessions lasting 30–60 min, exhibited the potential to induce a 35–60% increase in irisin levels [122,156,157,158,159,160,161,162,163].

### 3.15. Behavior of Exercise-Released Irisin with Tumors

Examining the previous section on Irisin reveals its intriguing connection with tumors. However, it is important to note that Irisin behaves differently in the presence of cancerous tumors. Initially, reliable clinical studies have indicated that specific solid tumors can lead to a decrease in Irisin levels. Specific solid tumors, particularly breast cancer and hepatocellular carcinoma, have been reported to exhibit significantly reduced circulating irisin levels compared with healthy individuals. In breast cancer patients, a ~40–50% decrease in serum irisin was demonstrated. Similarly, markedly lower irisin concentrations in virus-related liver cirrhosis and hepatocellular carcinoma cases were found [55,56,57]. These observations indicate that certain solid tumors suppress muscle-derived irisin production, potentially facilitating cancer-associated metabolic remodeling [53,58,59]. This decrease is attributed to the notion that an increase in Irisin can hinder tumor growth, which holds considerable significance for physically active individuals [53,58,59]. On the contrary, other articles have reported that in certain tumor types, an elevation in Irisin levels stimulates tumor growth and proliferation. Nevertheless, it remains challenging to definitively establish the effectiveness of exercise, accompanied by changes in Irisin levels (i.e., its increase), for cancer patients with solid tumors [53,58,59]. However, it appears that Irisin predominantly exerts suppressive effects.

### 3.16. IL-6 Response to Exercise

IL-6, a fascinating myokine, has captured the attention of researchers in relation to exercise. During exercise, skeletal muscle exhibits a remarkable ability to raise plasma IL-6 levels, surpassing baseline concentrations by more than 100 times [164,165]. This effect is particularly prominent in sports training that focuses on resistance exercises, which activate type 1 muscle fibers intensively, even at lower percentages of maximum force production [166,167,168]. Studies exploring the role of IL-6 have revealed that plasma IL-6 concentrations reach their peak either immediately after or shortly following the completion of exercise, with the duration of training exerting an influence on this peak response [169]. Building upon these findings, a study has indicated that performing acute resistance exercises at an intensity ranging from 70 to 85% 1RM, with a repetition range of 10–15 and 2–3 sets, can sustain elevated IL-6 levels for up to 4 h’ post-exercise [170]. Other investigations have also reported that engaging in resistance exercises at an intensity of 75–95% 1RM, with repetitions ranging from 8 to 12 and 3–5 sets, can induce an IL-6 increase that lasts for as long as 4, 8, or even 24 h after the training session [171,172,173,174,175]. The magnitude of this increase, observed to be between 16 and 32% in the reviewed studies, is primarily influenced by the type of muscle contraction. Researchers widely concur that the contraction type of muscle fibers strongly affects the release of IL-6 [164,165,166,167,168,171,172,173,174,175]. Furthermore, a study focusing on moderate-intensity exercise (6 × 5 min at 60% of heart rate reserve) has demonstrated an elevation in IL-6. Notably, this study revealed that aerobic exercise can potentially reduce the proliferation of colon cancer cells in vitro by regulating IL-6-induced DNA damage and repair [176]. Conversely, another study has shown that acute aerobic exercise at an intensity of 65–80% of maximum HR can also lead to an increase in IL-6 [177]. By comprehensively analyzing multiple studies, it becomes evident that aerobic exercises within the 65–90% range of maximum heart rate can substantially contribute to IL-6 elevation [174,178,179,180,181,182]. Nevertheless, this review of studies underscores an important point: the intensity of training plays a pivotal role in stimulating IL-6 secretion, as it directly impacts muscle fiber activation.

However, the chronic effects of exercise on IL-6 are important and should not be overlooked when drawing comprehensive conclusions. Epidemiological studies have reported an inverse association between resting IL-6 levels and regular physical activity [183,184]. Individuals engaged in long-term aerobic and resistance training have also shown marked reductions in resting IL-6 levels [185]. In addition, one study found that six months of aerobic exercise, performed three times per week at 70–80% of maximal heart rate, significantly reduced IL-6 levels [186]. Other studies have likewise shown that aerobic exercise performed at 50–65% of VO_2_ max and 55–75% of maximal heart rate for 30–50 min per session, three times per week, combined with resistance training at 55–85% of 1RM for 10–15 repetitions and 3–5 sets, reduced IL-6 by 16–53% [187,188,189,190,191,192,193,194]. However, IL-6 also exhibits a transient increase after individual exercise sessions during chronic training programs [195]. Overall, exercise appears to enhance the synthesis and release of IL-6 from contracting skeletal muscle. By contrast, short-duration or low-intensity exercise may not elicit the same systemic IL-6 response [196].

### 3.17. Behavior of Exercise-Released IL-6 with Tumors

In the previous section, we discussed the important role of IL-6 in the TME. Extensive research has shown that increased levels of IL-6 can significantly contribute to metastasis, tumor growth and proliferation in the body [63,64]. Moreover, this increase in IL-6 can induce a potent state of inflammation within the tumor [63,64]. However, when it comes to exercise, an intriguing finding has emerged: chronic resistance and aerobic exercises can lead to a reduction in IL-6 levels by triggering physiological adaptations in the body. Nevertheless, even after engaging in long-term training, there is still an observed rise in IL-6, which could potentially benefit the tumor. This increase becomes particularly evident during acute exercise sessions. Consequently, Fisher’s study suggests that low-intensity and short-duration exercises may only result in minimal reductions in IL-6. It is important to note that we cannot definitively conclude that exercise accompanied by an IL-6 response is suitable for solid tumors, as we observe an immediate increase in IL-6 levels following exercise.

In Fisher’s meta-analysis [196] the author aggregated data from controlled trials investigating acute and chronic exercise interventions and their influence on circulating IL-6. Using a Fisher-type synthesis approach, the study combined independent *p*-values derived from multiple experiments to estimate an overall effect size across heterogeneous protocols. This statistical integration allowed comparison of IL-6 dynamics between varying intensities and durations of exercise. The findings indicated that low-intensity (<55% VO_2_ max) and short-duration (<30 min) sessions yield only modest decrements in resting IL-6, whereas prolonged (>60 min) or high-intensity (>70% VO_2_ max) paradigms drive substantial acute IL-6 elevation followed by long-term adaptive reduction. Fisher’s cumulative analysis therefore highlighted that the magnitude of IL-6 change depends more on total physiological load and fiber recruitment than on exercise modality.

### 3.18. FGF-21 Response to Exercise

FGF-21 has captured the curiosity of researchers as an intriguing myokine. However, the limited studies on its acute response to resistance exercise make it challenging to arrive at definitive conclusions. Nevertheless, some studies suggest that resistance training with an intensity of 70–95% of 1RM, involving 8–10 repetitions and 3–5 training sets, potentially contributes to an elevation in FGF-21 levels [91,197,198,199]. Interestingly, Tanimura et al.’s (2016) reveals that aerobic exercise at 70% of maximal oxygen consumption (VO_2_ max) for 60 min can induce an increase in FGF-21 concentration [200]. Cuevas-Ramos conducted a study demonstrating that training at 75% of VO_2_ max, lasting 60 min per session, three times a week for two weeks, leads to an elevation in FGF-21 levels [201]. Kim et al. also support these findings, reporting that running on a treadmill for 30 min at 50% or 80% of VO_2_ max acutely raises FGF-21 levels [202]. Furthermore, other studies indicate that training with an intensity ranging from 65% to 75% of VO_2_ max, lasting between 30 and 60 min, effectively increases FGF-21 [203,204,205,206]. These studies generally observe FGF-21 increases of 6% to 21% based on their respective results [203,204,205,206].

In accordance with studies examining acute responses, the inclusion of studies investigating chronic responses can contribute to drawing more robust conclusions in this section. Jokar et al. (2013) conducted a study examining the effects of 12 weeks of resistance training using two different intensities: 80% of 1RM for 10 repetitions and 3 sets, and 60% of 1RM for 20 repetitions and 3 sets [199]. The researchers reported that both intensities led to a reduction in FGF-21, but resistance training at 80% of 1RM had a greater impact on this reduction [199]. Another study reported a decrease in FGF-21 following 12 weeks of resistance training, performed 3 days per week, with 10 repetitions at 70% of 1RM [91]. Fereidoonfar et al. in 2020 also supported these findings in their study, concluding that 8 weeks of resistance training with an intensity ranging from 50% to 70% of 1RM, 10 to 16 repetitions, and 3 sets performed in 3 sessions per week can lead to a decrease in FGF-21 [207]. Consistent with these studies, other research has indicated that resistance exercises with intensities ranging from 50% to 80% of 1RM, 8 to 16 repetitions, 3 to 4 sets, and performed in 3 sessions per week for a duration of 6 to 14 weeks can contribute to the reduction in FGF-21 [208,209,210,211,212]. However, it should be noted that the reported reductions in FGF-21 across these studies ranged from 26% to 43% [91,199,207,208,209,210,211,212]. Additionally, studies employing alternative interventions have also reported on the effects of resistance exercises. For instance, one study suggested that engaging in aerobic exercise 3 times a week at 60% of maximum HR for 8 weeks can lead to a decrease in FGF-21 [213]. Another study found that 3 months of aerobic exercise, consisting of 45 min sessions performed at an intensity of 60–75% of maximum HR, 5 times per week, resulted in a reduction in FGF-21 [214]. Similarly, a study reported that incorporating high-intensity interval training (4  ×  4 min at 85–95% of HRmax with 3 min of active rest at 50–60% of HRmax in between intervals) and moderate-intensity continuous training (walking/running) for 47 min at 60–70% of HRmax, performed 3 times per week for 12 weeks, led to decreases in FGF-21 [215]. In a study by Taniguchi et al. (2015), it was found that aerobic exercise at an intensity of 60–70% of VO_2_ max, lasting 30–45 min, and performed 3 to 5 sessions per week for 5 weeks resulted in a decrease in FGF-21 [216]. Similarly, other studies have indicated that aerobic exercises with intensities ranging from 50% to 65% of VO_2_ max, durations of 30 to 50 min, performed 3 to 5 sessions per week for 4 to 12 weeks, can significantly contribute to the reduction in FGF-21 [198,215,217,218,219]. The extent of the reduction in FGF-21 across these studies ranged from 35% to 58% [198,213,214,215,215,216,217,218,219].

### 3.19. Myonectin Response to Exercise

Myonectin is a recently discovered myokine. Despite its novelty, the scarcity of dedicated investigations necessitates a comprehensive review of the available literature. One study found an 8-week resistance training regimen, conducted thrice weekly, with intensities of 50% to 70% and 75% to 90% of the one-repetition maximum (1RM), reduced myonectin levels [220]. Another study investigated an aerobic exercise, implementing a four-week protocol, five days per week, at intensities ranging from 50% to 65% of VO_2_ max, also led to a noticeable reduction in myonectin expression [221]. Another study looked into the effects of eight-week exercise regimen involving three weekly sessions of 45 min of aerobic exercise. This study incorporated progressive increments in running intensity, commencing at 50% of maximum heart rate in the initial two weeks, ascending to 60% in the subsequent week, further elevating to 65% in the succeeding week, and ending at 70% in the final two weeks. This study was also associated with a marked decline in myonectin levels [222]. Another study further supports that sustained engagement in aerobic exercises, extending between 5 and 10 weeks, at intensities between 55% and 70% of VO_2_ max, and durations ranging from 30 to 45 min, causes a reduction in myonectin levels [223,224,225,226,227,228]. Collectively, these findings highlight the potential of exercise as a modulator of myonectin dynamics, paving the way for future inquiries into this field of myokine research.

### 3.20. Behavior of Exercise-Released Myonectin with Tumors

Elevated levels of myonectin were found to have a stimulatory effect on tumor proliferation and growth, while also playing a pivotal role in the development of cancer-associated cachexia [72,73]. Nonetheless, the current body of knowledge surrounding myonectin’s impact on tumors remains limited, presenting a formidable challenge in comprehensively assessing its potential dangers. In contrast, a compelling body of research consistently demonstrates that chronic exposure to myonectin leads to a reduction in its levels [72,73]. This finding raises the possibility that modulating myonectin concentrations could hold significant therapeutic benefits for patients with solid tumors. By pursuing strategies to diminish myonectin, it may be plausible to mitigate the deleterious effects associated with tumor progression and cancer-induced muscle wasting, thereby improving the overall well-being and prognosis of affected individuals. Further investigations are warranted to find the underlying mechanisms of myonectin’s influence on tumor biology. Thus, it will not only deepen the understanding of the complex interplay between myonectin and tumorigenesis but also pave the way for the development of targeted interventions that exploit the therapeutic potential of myokines in the context of cancer management (Table 2A,B).

### 3.21. Tumor-Specific Immunomodulatory Roles of Myokines

The systemic immunomodulatory effects of exercise-induced myokines are highly contingent upon the specific tumor microenvironment (TME) of different cancer types. Rather than exerting a uniform anti-tumor effect, myokines act as context-dependent signaling molecules that recruit, activate, or reprogram immune cells within the TME. In Breast Cancer, myokines like IL-15 and Irisin primarily drive the recruitment and infiltration of Natural Killer (NK) cells and CD8+ cytotoxic T lymphocytes. IL-15 upregulates the expression of activating receptors (such as NKG2D) on NK cells, leading to direct tumor lysis [229,230,231,232]. In Colorectal Cancer, SPARC (Secreted Protein Acidic and Rich in Cysteine) plays a crucial role by binding to the extracellular matrix, which not only inhibits tumor cell proliferation but also promotes the infiltration of tumor-suppressing M1 macrophages over immunosuppressive M2 macrophages [233,234,235]. In Lung Cancer, transient spikes in muscle-derived IL-6 participate in a complex signaling axis; while chronic IL-6 is often pro-tumorigenic, acute exercise-induced IL-6 pulses act as a chemotactic factor, mobilizing NK cells to the tumor site and enhancing dendritic cell antigen presentation [236,237]. Conversely, in Pancreatic Cancer, myokines like LIF (Leukemia Inhibitory Factor) and Myostatin downregulation alter the dense desmoplastic stroma, facilitating better infiltration of CD8+ T cells into an otherwise immunologically ‘cold’ tumor [238,239]. These distinct tumor-specific pathways underscore the complexity of myokine-mediated immunomodulation and emphasize the need for cancer-specific exercise prescriptions (Table 3).

**Table 3:** Tumor-specific immunomodulatory mechanisms of exercise-induced myokines across various cancer types.

Tumor Type	Key Myokine(s) Involved	Primary Immunomodulatory Mechanism	Key Immune Cell Targets
Breast Cancer	IL-15, Irisin	Enhances cytolytic activity; upregulates NKG2D receptors; promotes tumor homing of effector cells.	NK cells, CD8+ T cells
Colorectal Cancer	SPARC, IL-6 (Acute)	Inhibits Wnt/β-catenin signaling; promotes M1 macrophage polarization; suppresses pro-tumor inflammation.	M1 Macrophages, NK cells
Lung Cancer	IL-6 (Acute pulses), IL-15	Stimulates dendritic cell maturation; enhances trafficking of cytotoxic lymphocytes to the lung parenchyma.	Dendritic cells, NK cells, CD8+ T cells
Pancreatic Cancer	LIF, Decorin	Modulates stromal density; increases CD8+ T cell perfusion into the immunosuppressive tumor core.	CD8+ T cells, CAF (Cancer-Associated Fibroblasts)
Prostate Cancer	Oncostatin M (OSM), Irisin	Induces apoptosis via STAT3 pathway modulation; suppresses macrophage-mediated immunosuppression.	Macrophages, Cytotoxic T cells

## 4. Discussion

This review included studies that involved both preclinical and human (cancer and non-cancer) data, and although this is not a systematic review or meta-analysis, there are important generalizations that may be helpful in considering exercise prescriptions. Basic and clinical research fulfill complementary roles in exercise oncology, but they do not operate at the same level of translational readiness. Basic research is indispensable for dissecting the molecular basis of exercise-induced myokine signaling and its interactions with the tumor microenvironment. Nevertheless, the pleiotropic and context-dependent nature of myokines means that mechanistic data may be difficult to interpret and may not anticipate the direction or magnitude of clinical effects. Indeed, some preclinical findings may even raise concerns regarding tumor-promoting or pro-angiogenic signaling under specific conditions. Clinical research therefore remains essential, as it provides the most direct evidence regarding efficacy, safety, and therapeutic relevance, often before the mechanistic basis is fully elucidated. In this sense, clinical outcomes should be viewed as a critical guide for future mechanistic work rather than as a secondary confirmation of basic science findings.

### 4.1. Mechanistic Interplay Between Myokines and Cancer Biology

The interpretation of circulating myokine responses in the context of cancer cachexia is fundamentally challenged by the inherent heterogeneity of exercise-induced adaptations. Our synthesis highlights a critical dichotomy: while acute exercise—spanning modalities from resistance and endurance training to high-intensity interval training (HIIT)—often triggers transient surges in myokines such as IL-6 and irisin, these immediate responses do not inherently predict long-term resting adaptations. As evidenced by the variability across diverse study designs and intervention durations, the current lack of standardized exercise prescription remains a primary barrier to establishing a coherent translational framework [11,76,240]. This underscores the necessity for future clinical trials to move beyond descriptive reporting and prioritize the synchronization of training protocols, exercise intensity, and the temporal alignment of outcome measurements. By systematically addressing these protocol-dependent nuances, this review provides a more rigorous roadmap for developing exercise-based therapeutics that are physiologically sound and clinically reproducible [11,76,240]. Building upon our previous conceptual framework regarding the broad spectrum of exerkines in cancer cachexia Ahmadi Hekmatikar et al. (2023) [11], the current review refines this scope. While our previous work established the systemic importance of exercise-mediated signaling in cachectic patients, the current study narrows the focus to myokines specifically. This specialization allows for a more granular mechanistic examination of how exercise-induced skeletal muscle signaling directly interacts with tumor-associated pathways—a critical transition from the systemic perspective of exerkines to the mechanistic resolution of myokines in cachexia management.

Although myokines influence multiple physiological systems—including inflammation, metabolism, and immune function—muscle wasting remains the most clinically relevant outcome of their dysregulation in cancer. As such, the interaction between tumor-derived signals and myokine-mediated muscle loss provides the biological foundation for interpreting exercise effects in oncology. Although the relationship between physical activity and cancer is increasingly acknowledged, the mechanistic underpinnings remain incompletely understood. This narrative review set out to explore how exercise—particularly via myokines—may influence the TME and cancer-related muscle wasting. Myokines have a close association with cancer, and it appears that during solid tumor growth, the tumor’s physiology induces detrimental changes in myokines, leading to muscle wasting known as cancer cachexia [24,227]. Epidemiological evidence indicates that skeletal muscle loss is prevalent in most cancer types, with solid tumors exhibiting the highest occurrence [228,229]. Skeletal muscle is more severely affected by cachexia in cancer compared to other organs [228,229]. It is now well-established that solid tumors (STs) induce systemic inflammation, contributing to the dysregulation of myokine profiles, most notably increased levels of IL-6, irisin, myostatin, and FGF-21, and decreased follistatin and myonectin. These changes are strongly implicated in the pathophysiology of cancer cachexia, a multifactorial syndrome marked by progressive muscle wasting and metabolic dysfunction [18,28,241,242]. Emerging evidence highlights that tumor-induced inflammation activates key catabolic pathways in skeletal muscle, such as the ubiquitin–proteasome system, where Atrogin-1 and MuRF-1 are central players in muscle protein degradation [243]. These pathways are directly or indirectly modulated by circulating myokines, suggesting that exercise-induced counterregulation of these molecules may have therapeutic potential [243]. However, it remains unclear how individual myokines mechanistically alter tumor cell behavior, immune responses, or metabolism across different cancer types. Beyond their systemic and paracrine effects on tumor physiology, myokines play a fundamental role in the regulation of skeletal muscle stem cells (satellite cells), which are essential for muscle repair and regeneration. Exercise-induced release of myokines such as IL-6, irisin, and FGF-21 activates satellite cell-related signaling through JAK/STAT3, AMPK–PGC1α, and PI3K/Akt cascades, respectively. These pathways enhance proliferation and differentiation of muscle progenitor cells, maintaining muscle integrity and counteracting cancer-associated cachexia. Conversely, overexpression of myostatin inhibits satellite cell activation by repressing MyoD/MyoG expression, thereby aggravating muscle wasting observed in advanced tumors. Regulation of these muscle stem cell dynamics not only determines the host’s physical resilience but may indirectly modulate the TME by altering cytokine composition and metabolic substrate availability. Integrating stem-cell physiology into myokine research thus provides a more comprehensive understanding of how exercise-mediated molecular adaptations influence both muscle homeostasis and tumor progression.

### 4.2. Cachexia and Myokine Regulation Through Exercise

Cachexia is very common in patients with solid tumors and is closely associated with dysregulated myokine signaling. Several studies have shown that exercise can modulate this response. For example, chronic moderate-intensity aerobic and resistance training have consistently been shown in healthy individuals to reduce circulating myostatin and increase follistatin levels—adaptations that support muscle hypertrophy and counteract catabolic signaling. In cancer patients, tumor-derived factors typically elevate baseline myostatin and suppress follistatin, thereby contributing to muscle wasting. Nevertheless, the available clinical and translational studies indicate that regular exercise can partially reverse these tumor-driven alterations by lowering myostatin and increasing follistatin, ultimately attenuating cancer-related muscle wasting. These effects are generally smaller and more variable than in healthy populations but remain physiologically meaningful [11,12]. In addition, irisin, a myokine released during endurance exercise, may influence tumor cell apoptosis and energy metabolism [11,12]. As shown in Table 2A, changes in myokines are observed at different intensities. Nevertheless, despite promising preclinical data, the mechanistic links are poorly defined. Most studies to date have relied on animal models or laboratory systems and have limited translational evidence in clinical populations.

### 4.3. Acute vs. Chronic Effects of Exercise

The distinction between acute and chronic exercise responses is critical. Our review indicates that chronic exercise, particularly at moderate intensity, induces more stable and beneficial myokine profiles than single exercise bouts. Examples of effective chronic protocols include walking, swimming, cycling, and resistance exercises performed over 6 to 12 weeks, with durations of 30 to 60 min, three times per week. In contrast, acute high-intensity exercise may lead to transient elevations in inflammatory myokines such as IL-6, with potentially negative implications in certain tumor contexts [83,91,107,111,112,113,114,115,117,118,119,120,121,122,124,125,126,127,128,129,130,131,132,137,139,146,147,148,151,152,153,154,155,156,157,158,159,160,161,162,163,166,167,168,174,178,179,180,181,182,187,188,189,190,191,192,193,194,198,199,203,204,205,206,207,208,209,210,211,212,213,214,215,215,216,217,218,219,220,221,223,224,225,226,227,228,244]. Thus, the duration, intensity, and type of exercise should be carefully tailored, particularly during cancer treatment.

Importantly, the biological effects of exercise-induced myokines such as IL-6, irisin, and FGF-21 should be interpreted as context dependent rather than intrinsically beneficial or detrimental. Current evidence suggests that the transition between anti-tumorigenic and pro-tumorigenic actions is influenced by multiple interacting factors, including the circulating concentration and duration of myokine exposure, the modality, intensity, and chronicity of the exercise stimulus, receptor expression and downstream signaling pathways, as well as the biological characteristics of the tumor microenvironment. For example, transient elevations in IL-6 following acute exercise are generally associated with metabolic regulation and immune cell recruitment, whereas chronically elevated IL-6 concentrations associated with persistent systemic inflammation have been linked to tumor progression and cancer cachexia. Similar context-dependent mechanisms have been proposed for irisin and FGF-21, although the molecular thresholds governing these transitions remain poorly understood. Defining these biological thresholds will be essential for developing safe, individualized, and evidence-based exercise prescriptions in oncology.

### 4.4. Exercise Oncology and Chemotherapy Adjacency: Mechanistic and Demographic Nuances

Beyond attenuating muscle wasting, exercise training serves as a potent adjuvant to standard oncological therapies by directly modulating chemotherapy efficacy and tolerability. During exercise, the systemic release of myokines—such as IL-15, IL-6, and irisin—coordinates with increased cardiac output to drive tumor vascular remodeling [11,13,14,24,25,30,76,78,79,133,134,196,240,245,245,246,247,248,249,250,251,252]. This transient normalization of the tumor microvasculature enhances intratumoral perfusion, thereby facilitating a more efficient delivery of chemotherapeutic agents and reducing hypoxia-induced chemoresistance. Furthermore, myokine-mediated activation of natural killer (NK) cells and CD8+ T-lymphocytes enhances immune surveillance, creating a synergistic anti-tumor microenvironment during active chemotherapy cycles [11,13,14,24,25,30,76,78,79,133,134,196,240,245,245,246,247,248,249,250,251,252].

Crucially, these exercise-induced adaptations are not uniform and are heavily influenced by biological sex and age. Sex-specific differences in hormone profiles (specifically the ratio of circulating estrogen to testosterone) and skeletal muscle phenotype (type I versus type II fiber distribution) dictate both the baseline myokine reservoir and the magnitude of their release post-exercise [11,13,14,24,25,30,76,78,79,133,134,196,240,245,245,246,247,248,249,250,251,252]. For instance, females typically exhibit distinct baseline levels and post-exercise kinetics of myokines like adiponectin and IL-6 compared to males, which may alter the systemic anti-cachectic signaling cascade. Similarly, age-specific factors, such as sarcopenia and chronic low-grade inflammation (inflammaging), alter the responsiveness of senescent muscle tissue [11,13,14,24,25,30,76,78,79,133,134,196,240,245,245,246,247,248,249,250,251,252]. Aging populations often exhibit a blunted anabolic and myokine secretory response (anabolic resistance) to acute exercise, necessitating tailored, higher-intensity or progressive volume-based exercise prescriptions to achieve comparable therapeutic outcomes to younger counterparts. Acknowledging these demographic variations is essential for transitioning from a ‘one-size-fits-all’ exercise prescription to individualized, precision exercise oncology [11,13,14,24,25,30,76,78,79,133,134,196,240,245,245,246,247,248,249,250,251,252].

### 4.5. Exercise Intensity and Evidence-Based Exercise Recommendations

General exercise guidelines (e.g., 150 min of aerobics or 75 min of anaerobic training per week) are helpful but insufficiently specific in oncology settings. Our review underscores that moderate-intensity exercise (e.g., 55–75% HRmax, Borg scale 4–6/10) yields the most consistent myokine responses associated with anti-inflammatory and anti-cachectic effects. For resistance training, protocols involving 8–15 repetitions and multiple sets appear to be optimal for chronic myokine modulation [83,91,107,111,112,113,114,115,117,118,119,120,121,122,124,125,126,127,128,129,130,131,132,137,139,146,147,148,151,152,153,154,155,156,157,158,159,160,161,162,163,166,167,168,174,178,179,180,181,182,187,188,189,190,191,192,193,194,198,199,203,204,205,206,207,208,209,210,211,212,213,214,215,215,216,217,218,219,220,221,223,224,225,226,227,228]. Nevertheless, there is a lack of consensus across studies regarding the ideal prescription, and evidence is often heterogeneous, with studies differing in populations, cancer types, intervention designs, and outcome measures.

Importantly, the molecular responses induced by acute exercise differ substantially from those elicited by long-term exercise training. A single exercise bout may induce transient increases in several exercise-responsive myokines, including IL-6, which functions as a physiological signaling molecule involved in metabolic regulation and immune cell recruitment rather than as a marker of chronic inflammation. In contrast, regular exercise training promotes sustained physiological adaptations characterized by reduced basal inflammatory status, increased skeletal muscle mass, decreased resting IL-6 concentrations, and increased expression of anabolic myokines such as follistatin. These chronic adaptations are likely to provide greater protection against cancer-associated cachexia and should therefore be considered when designing individualized exercise programs for patients with cancer.

Based on the evidence synthesized in the present review, Table 4 summarizes the exercise prescription parameters that have been most consistently associated with favorable myokine responses in the context of cancer. Given the heterogeneity of the available studies, these recommendations should not be interpreted as definitive clinical guidelines but rather as an evidence-informed framework to guide future research and support individualized exercise prescription. Whenever possible, the recommendations distinguish between interventions primarily aimed at attenuating cancer cachexia and those with potential anti-tumor effects, recognizing that the latter remain supported predominantly by preclinical evidence.

### 4.6. Gaps and Inconsistencies in the Literature

While the overall trend points toward beneficial effects of structured exercise on myokines and cancer physiology, several gaps and inconsistencies remain:

- Many studies lack standardized protocols for exercise intensity and duration.
- There is limited high-quality clinical data confirming preclinical findings.
- Most studies do not account for cancer stage, treatment phase, or individual patient variability.
- Mechanistic pathways (e.g., JAK/STAT, AMPK-PGC1α, PI3K/Akt) are mentioned but rarely investigated in depth.

Recent evidence suggests these signaling routes represent key molecular links between exercise and tumor adaptation. Expanding future studies to quantify pathway activation—such as STAT3 phosphorylation for IL-6, PGC-1α upregulation for irisin, and Akt/mTOR modulation for myonectin—could refine mechanistic modeling and explain tumor-specific differences in myokine responses. These gaps hinder the formulation of precise, evidence-based exercise recommendations for cancer patients.

### 4.7. Limitations of Current Literature and Future Directions

Despite the promising therapeutic potential of exercise-induced myokines in cancer cachexia, several critical limitations across the existing literature must be acknowledged to guide future translational research.

First, a substantial proportion of the mechanistic data linking myokine signaling to tumor suppression and muscle preservation is derived from preclinical rodent models and in vitro studies. Although these experimental models provide valuable mechanistic insights, many of the molecular pathways discussed in this review—including several interactions between exercise-induced myokines and the tumor microenvironment—have not yet been confirmed in well-designed human studies. Consequently, caution is warranted when translating these findings into clinical exercise recommendations, and further clinical investigations are required to validate their physiological relevance and therapeutic potential. Moreover, translating these findings to clinical oncology remains highly challenging due to species-specific variations in myokine secretion kinetics, metabolic rates, and tumor microenvironment dynamics.

Second, there is a profound lack of temporal and methodological standardization in clinical studies. Myokines are characterized by rapid clearance and short biological half-lives, yet blood sampling time points vary drastically across protocols (ranging from immediately post-exercise to several days post-intervention). This inconsistency makes it difficult to distinguish between acute, transient spikes and chronic, systemic adaptations. Third, the confounding influence of ongoing oncological treatments is frequently underreported. Chemotherapeutic agents and radiation therapy induce severe systemic toxicity and skeletal muscle degradation (chemo-induced sarcopenia), which dynamically alters the muscle’s secretory capacity. How different chemotherapy regimens interact with acute exercise-induced myokine responses remains poorly characterized in human cohorts. Lastly, significant variability exists regarding measurement essays. The use of different detection methods (e.g., enzyme-linked immunosorbent assay [ELISA] versus multiplex bead-based assays) across laboratories introduces discrepancies in quantifying absolute circulating myokine concentrations, particularly for low-abundance proteins, hindering cross-study comparisons.

### 4.8. A Look at Future Studies

This review has outlined several critical gaps in our current understanding of how myokines—exercise-induced signaling molecules—interact with tumor biology. To advance the field, future research should prioritize three main directions. First, the standardization of exercise protocols—including duration, intensity, and modality—is essential to enable cross-study comparison and identification of reproducible dose–response patterns. Second, studies should expand beyond well-characterized myokines such as IL-6, irisin, and FGF-21 to include emerging molecules like myonectin, decorin, apelin, and β-aminoisobutyric acid, whose oncologic roles remain poorly defined. Third, establishing integrative designs that simultaneously measure myokine dynamics and clinical outcomes—such as muscle mass, inflammatory biomarkers, and tumor regression—will provide translational insight bridging molecular and physiological effects. Together, these priorities will help refine exercise-based interventions as legitimate adjuvant strategies in cancer therapy. Notably, there is a lack of robust data on how different exercise modalities, particularly acute versus chronic regimens, influence myokine profiles in the context of solid tumors. Addressing this gap requires standardized experimental protocols that can distinguish between transient and long-term adaptations in myokine expression and their downstream effects on tumor progression.

Among the less studied myokines, myonectin has emerged as a potentially important player. While preliminary evidence suggests its role in metabolic regulation, its specific effects on tumor physiology and cancer cachexia remain poorly understood. Future investigations should prioritize mechanistic studies that examine how myonectin responds to exercise and interacts with the tumor microenvironment. In addition, well-designed clinical studies are warranted to determine its translational relevance as a potential biomarker and therapeutic target for exercise-based interventions aimed at attenuating cancer-associated cachexia. Integrating findings from both preclinical models and human studies will be essential to clarify the biological and clinical significance of myonectin in exercise oncology.

In addition, expanding the research beyond myokines to include other exerkines—such as adipokines and hepatokines—may offer a more integrative perspective on the systemic effects of exercise in cancer settings. Methodological rigor and cross-disciplinary collaboration will be essential to address these complex biological interactions and to bridge the gap between laboratory findings and clinical application.

We have reported that myokines (irisin and FGF-21) have shown both tumor suppressive and proliferative effects. Myokines can have both tumor suppressive and proliferative effects depending on the specific myokine and the type of cancer. While many myokines have shown tumor suppressive activity by inhibiting cancer cell proliferation, promoting apoptosis, and limiting metastasis, some may also contribute to tumor progression under certain circumstances. This review could help understand the specific roles of different myokines in cancer and could lead to the development of novel exercise-based therapeutic strategies targeting myokine pathways to inhibit tumor growth or control cancer-associated cachexia. Further research is needed to: Fully elucidate the complex interplay between different myokines, their effects on various cancer types, and the type of exercise implemented; To determine the precise mechanisms by which myokines influence cancer cell behavior and the TME in the context of physical exercise; to develop targeted therapies based on myokine modulation for cancer treatment that utilize therapeutic exercise as an adjuvant to therapeutic therapy. A major limitation in the current literature is the lack of standardized exercise protocols regarding training volume, intensity, and progression. In exercise oncology, interventions must transition from generic physical activity recommendations to precise, individual clinical prescriptions. As emphasized in the foundational work by Winters-Stone et al. [insert reference], exercise trials in cancer populations should adhere to the core principles of training theory. This requires not only detailed reporting of the FITT components (frequency, intensity, time, and type) but also systematic application of training concepts such as specificity, progressive overload, progression, baseline initial values, diminishing returns, and reversibility. Standardizing these training parameters is essential to establish dose–response relationships for myokine secretion and to design effective therapeutic exercise regimens [11,14,245,246,247,249,252,253].

A primary frontier for future research is the systematic mapping of how distinct exercise modalities (e.g., HIIT, resistance training, or concurrent protocols) differentially modulate the myokine-immune axis within the tumor microenvironment (TME). Current evidence suggests that myokines do not act in isolation but serve as primary drivers of immune cell trafficking and activation. However, the specific ‘exercise dose’ required to optimize the recruitment of natural killer (NK) cells and CD8+ T cells via myokine signaling (such as IL-15 and SPARC) remains a critical knowledge gap. Future investigations should prioritize longitudinal designs that correlate specific training variables—intensity, volume, and mechanical loading—with systemic immunomodulatory profiles. Specifically, research must determine whether acute, high-intensity bouts provide a superior ‘immunogenic spike’ compared to chronic, moderate-intensity adaptations for tumor suppression. Furthermore, identifying the ‘minimal effective dose’ of exercise capable of reversing immune exhaustion (e.g., PD-1/PD-L1 signaling modulation) through myokine-mediated pathways could revolutionize adjuvant cancer care. Additionally, the integration of high-resolution immunophenotyping with myokine kinetic profiling will be essential. Understanding how the temporal release of myokines during different phases of an exercise program influences the polarization of M1/M2 macrophages or the infiltration of regulatory T cells (Tregs) will provide the mechanistic foundation for precision exercise prescription. This trajectory—moving from general physical activity to protocol-specific immunomodulation—is the key to establishing exercise as a targeted biological therapy in clinical oncology.

Furthermore, bridging the current gap in molecular oncology requires the strategic integration of high-throughput multi-omics platforms, including genomics, transcriptomics, proteomics, and metabolomics. Currently, the literature on exercise-induced myokine kinetics remains characterized by fragmented, single-protein analyses that fail to capture the systemic complexity of the muscle-tumor secretome. Future investigations should leverage longitudinal transcriptomic and proteomic profiling of both skeletal muscle biopsies and circulating extracellular vesicles post-exercise. Mapping these comprehensive molecular networks will not only elucidate the complex, synergistic signaling pathways within the tumor microenvironment but also identify novel genomic signatures that predict individual patient responsiveness to exercise-induced immunomodulation. Transitioning to such systems-biology approaches is critical to establishing robust, data-driven targets for precision exercise oncology.

## 5. Conclusions

This structured narrative review synthesized current evidence on how exercise-induced myokines modulate the TME. Although consistent patterns were observed—such as IL-6’s dual immunomodulatory role, irisin’s pro-apoptotic effects, and myostatin’s downregulation favoring anabolic signaling—these findings remain largely qualitative and derived from limited observational and preclinical studies. Therefore, the conclusions should be interpreted cautiously. The present evidence supports a potential modulatory influence of moderate-intensity exercise on myokine profiles and related tumor physiology; however, causal relationships cannot be established due to the absence of quantitative synthesis and high variance among study designs. Rigorous clinical trials and meta-analytic approaches are needed to define dose–response relationships and to validate whether myokine modulation contributes directly to tumor control or systemic benefits in cancer patients. Exercise-mediated modulation of specific myokines represents one of the most promising mechanistic links between exercise and cancer biology. Among these, irisin and FGF-21 emerge as central molecules with dual yet context-dependent roles: irisin exerts pro-apoptotic and metabolic regulatory effects that may limit tumor progression, while FGF-21 contributes to energy balance and can display either protective or proliferative behavior depending on exercise intensity and tumor type. IL-6 remains a pivotal but complex cytokine, balancing inflammatory and immune-regulatory signaling. Collectively, these myokines illustrate how regular moderate-intensity exercise—integrating aerobic and resistance components—can promote anti-inflammatory and anti-cachectic effects and potentially modulate the tumor environment. Translational efforts should now focus on validating these molecular patterns in clinical populations and defining reproducible exercise prescriptions that harness their beneficial signaling. By aligning mechanistic insights with targeted training protocols, exercise may serve not merely as supportive care but as an adjuvant therapeutic avenue complementing conventional oncologic treatments. Although claims regarding tumor suppression or reduction in tumor size remain preliminary, several clinical trials have begun to evaluate this possibility. For instance, moderate aerobic and resistance training interventions in breast and colorectal cancer survivors have demonstrated improvements in systemic inflammation, IL-6 regulation, and quality of life, yet without significant changes in tumor dimensions. Similarly, lung and prostate cancer studies reported metabolic benefits and stabilization of tumor progression rather than measurable shrinkage. Collectively, existing clinical evidence indicates that while exercise may beneficially influence biomolecular and immunometabolic pathways involved in tumor control, confirmation of direct tumor-size reduction still requires large-scale, multi-center randomized controlled trials with standardized exercise protocols.

Importantly, the biological effects of exercise-induced myokines should not be considered universal but rather context-dependent, varying according to the tumor type, the characteristics of the tumor microenvironment, and the nature of the exercise stimulus. Consequently, future exercise-based therapeutic strategies should incorporate personalized exercise prescriptions that account for these factors to maximize their anticancer and anti-cachectic potential.

## Figures and Tables

**Table 1:** Summary of exercise-related regulation of myokines in cancer models, specifying direction of change (↑/↓) in circulating serum levels or tissue expression.

Skeletal Muscle	Cancer Type/Model	Direction (↑/↓)	Sample Type	Baseline Level (Healthy/Control)	Level in Cancer	Effects of Behavioral Changes
Myostatin	Cachexia, Sarcoma	↓ (muscle expression)	Muscle tissue	10–15 pg/mL	25–60 pg/mL	Migration of tumor-associated macrophages into the tumor and contributing to tumor proliferation and growth. It also leads to muscle atrophy and cancer cachexia.
Follistatin	Prostate, Hepatic	↑ (circulating)	Serum	150–200 ng/mL	180–240 ng/mL	It can lead to metastasis and angiogenesis around the tumor. It seems that it can play a significant role in tumor aggressiveness.
Irisin	Breast, Colon, Pancreatic	↑ (circulating)	Serum	45 ± 12 ng/mL	25 ± 10 ng/mL	Irisin shows a different behavior in relation to the tumor. In some tumors, it can lead to tumor proliferation and growth, and in others, it can lead to tumor suppression.
Interleukin-6	Breast, Lung, Liver	↑ (circulating serum)	Serum	200 ± 40 pg/mL	280 ± 50 pg/mL	Interleukin-6 in solid tumors can lead to tumor proliferation and growth. Finally, this proliferation can bring an aggressive tumor to the body.
FGF-21	Thyroid, Prostate	↑/↓ intensity-dependent	Serum	80–100 pg/mL	90–230 pg/mL	Like irisin, FGF-21 acts like a double-edged sword. It seems that in most studies, increasing FGF-21 can lead to tumor proliferation and growth and is effective in tumor metastasis. In some other studies, it seems that FGF-21 can lead to tumor suppression and prevent its growth.
Myonectin	Breast, Cachexia	↓ (serum and tissue)	Serum	↑ 12–18 ng/mL	6–9 ng/mL	Myonectin’s mechanism of action is not clear, but it seems to be indirectly related to tumor and its increase has been seen in cancer cachexia.

Abbreviations: ↑ = increase; ↓ = decrease; unless otherwise stated, changes refer to circulating (serum/plasma) concentrations measured after exercise. “Expression” indicates gene or protein levels quantified in muscle or tumor tissue. References provided correspond to primary experimental or review data.

**Table 2:** Myokine response to acute and chronic exercise.

(**A**)						
Exerkines Myokines (Skeletal muscle)	Resistance exercise Acute			Resistance exercise Chronic		
	60 to 90% 1RM OR Borg scale 5–9/10			45 to 70% 1RM OR Borg scale 2–4/10		
	Increase	Decrease	not clear	Increase	Decrease	not clear
Myostatin	✓				✓	
Follistatin	✓			✓		
irisin	✓	✓			✓	
IL-6	✓			✓	✓	
FGF-21	✓	✓			✓	
Myonectin			✓		✓	
Abbreviations = RM: repetition maximum; Borg scale: Borg Rating of Perceived Exertion; IL-6: interleukin 6; FGF - 21: fibroblast growth factor 21; Not clear: The available results are ambiguous or there is no information related to it.						
(**B**)						
Exerkines Myokines (Skeletal muscle)	Aerobic exercise Acute			Aerobic exercise Chronic		
	60–95% VO_2_ max OR 75–90% HRmax OR Borg scale of 6–10/10			45–65% VO_2_ max OR 55–75% HRmax OR Borg scale of 4–6/10		
	Increase	Decrease	not clear	Increase	Decrease	not clear
Myostatin	✓				✓	
Follistatin	✓			✓		
irisin	✓	✓			✓	
IL-6	✓				✓	
FGF-21	✓	✓			✓	
Myonectin		✓			✓	
Abbreviations = HR: heart rate; Borg scale: Borg Rating of Perceived Exertion; VO_2_ max: maximum volume of oxygen uptake; IL-6: interleukin 6; FGF - 21: fibroblast growth factor 21; Not clear: The available results are ambiguous or there is no information related to it.						

**Table 4:** Evidence-based exercise recommendations for modulating myokine responses in cancer patients based on the current literature.

Clinical Objective	Exercise Modality	Recommended Intensity	Frequency	Duration	Main Expected Myokine Responses	Level of Evidence
**Prevention/attenuation of cancer cachexia**	Moderate aerobic + resistance training	55–75% HRmax (Borg 4–6/10)	3 sessions/week	30–60 min for ≥6–12 weeks	↓ Myostatin, ↑ Follistatin, ↑ Irisin, improved anti-inflammatory profile	Moderate
**Maintenance of skeletal muscle mass**	Progressive resistance training	Moderate	2–3 sessions/week	8–15 repetitions, multiple sets	↑ Muscle protein synthesis, ↓ catabolic signaling	Moderate
**Potential support of anti-tumor immune responses**	Moderate aerobic exercise	Moderate	Regular long-term training	≥150 min/week	Transient ↑ IL-6, ↑ IL-15, ↑ NK-cell mobilization	Low–Moderate
**Acute exercise responses**	High-intensity exercise	High	Single bout	Variable	Transient myokine elevations; clinical significance remains uncertain	Low

The recommendations summarized in this table are based on the best currently available evidence and should not be interpreted as formal clinical guidelines. They are intended to provide a practical framework for future clinical studies and individualized exercise prescription in oncology. ↓ decrease, ↑ increase.

## Data Availability

No new data were created or analyzed in this study. Data sharing is not applicable to this article.
